# Psgl-1 Deficiency is Protective against Stroke in a Murine Model of Lupus

**DOI:** 10.1038/srep28997

**Published:** 2016-06-30

**Authors:** Hui Wang, Jason S. Knight, Jeffrey B. Hodgin, Jintao Wang, Chiao Guo, Kyle Kleiman, Daniel T. Eitzman

**Affiliations:** 1 University of Michigan, Department of Internal Medicine, Cardiovascular Research Center, Ann Arbor, Michigan, USA; 2 University of Michigan, Department of Internal Medicine, Division of Rheumatology, Ann Arbor, Michigan, USA; 3 University of Michigan, Department of Pathology, Ann Arbor, Michigan, USA

## Abstract

Systemic lupus erythematosus (SLE) is associated with an elevated risk of vascular complications, including premature stroke. Therapies targeting leukocyte recruitment may be beneficial in reducing vascular complications associated with SLE. Lupus was induced in female wild-type (WT) and P-selectin glycoprotein ligand-1 deficient (*Psgl-1*^−/−^) mice with pristane. Stroke was induced following photochemical injury to the middle cerebral artery (MCA). Stroke size was increased in pristane-treated WT mice compared to non-pristane-treated WT controls. However, stroke size was not increased in pristane-treated *Psgl-1*^−/−^ mice compared to controls, despite evidence of increased nephritis in *Psgl-1*^−/−^ mice. Pristane-treated WT mice showed elevated anti-dsDNA, anti-snRNP, CXCL1, and MCP-1 levels compared to untreated mice; however levels of anti-snRNP, MCP-1, and CXCL1 were reduced in pristane-treated *Psgl-1*^−/−^ mice compared to pristane-treated WT mice. Infiltration of neutrophils and macrophages at the cerebral infarction site were reduced in pristane-treated *Psgl-1*^−/−^ mice compared to pristane-treated WT mice. In conclusion, the increase in stroke size associated with lupus is prevented by Psgl-1 deficiency while nephritis is exacerbated. Therapies targeting Psgl-1 may be useful in the management of SLE patients at high risk of acute vascular complications although elucidation of downstream pathways will be necessary to identify targets that do not promote nephritis.

Systemic lupus erythematosis (SLE) is an autoimmune disorder associated with premature vascular disease, including stroke^1^,^2^,^3^,^4^. The heightened macrovascular risk is not fully explained by conventional risk factors for vascular disease^5^,^6^, and does not necessarily correlate with other autoimmune features of lupus. Pathways involving tissue leukocyte recruitment and activation may play important roles and serve as therapeutic targets related to vascular complications associated with SLE^7^,^8^. P-selectin glycoprotein ligand-1 (Psgl-1) is a leukocyte ligand that regulates recruitment and activation of multiple cell types through interactions with selectins^9^. The purpose of this study was to test the effect of pristane-induced lupus in a stroke model and to determine the effect of Psgl-1 inhibition on features of lupus and stroke size in mice.

## Methods and Materials

### Animals

Female C57BL6/J wild-type (WT) and Psgl-1 deficient (*Psgl-1*^−/−^) mice were originally purchased from Jackson Laboratory (Bar Harbor, Maine). *Psgl-1*^−/−^ mice were backcrossed to the C57BL6/J strain >16 generations before use in these experiments. Mice were housed under specific pathogen-free conditions in static microisolator cages with tap water ad libitum in a temperature-controlled room with a 12:12-hour light/dark cycle and were fed a standard laboratory rodent diet (No. 5001, TestDiet, Richmond, IN). All animal use protocols complied with the Principles of Laboratory and Animal Care established by the National Society for Medical Research and were approved by the University of Michigan Committee on Use and Care of Animals.

### Induction of lupus

To induce lupus, female WT and *Psgl-1*^−/−^ mice received an intraperitoneal injection of 0.5 ml pristane (2,6,10,14-tetramethylpentadecane) (Sigma, St Louis, MO) at 10 weeks of age as previously described^10^,^11^. PBS was administrated to control female WT and *Psgl-1*^−/−^ mice. Serum samples were collected via retro-orbital bleeding using capillary tubes 32 weeks following administration of PBS or pristane. Proteinuria was evaluated by urine albumin-to-creatinine ratio measured with the Albuwell M Test kit and Creatinine Companion (Exocell, Philadelphia, PA) as previously described^8^. 32 weeks after pristane, blood pressure was measured in non-anesthetized mice by tail plethysmography using the CODA Tail-Cuff Blood Pressure System (KENT Scientific Co., Torrington, CT) following manufactures’ instructions. For blood pressure measurements, mice were trained for seven consecutive days starting at 31 weeks following PBS or pristane treatment. Blood pressures were then determined at 32 weeks following treatment. All blood pressure measurements were consistently performed in the morning.

### Measurements of serum samples

At 32 weeks following pristane treatment, circulating concentrations of anti-double-stranded DNA antibodies (anti-dsDNA) (Alpha Diagnostic, San Antonio, TX), small nuclear ribonucleoprotein antibody (anti-snRNP) (Alpha Diagnostic, San Antonio, TX), chemokine (C-X-C motif) ligand 1 (CXCL1) (R&D Systems, Minneapolis, MN), interleukin-6 (IL-6) (R&D Systems, Minneapolis, MN), chemokine (C-C motif) ligand 3(CCL3) (R&D Systems, Minneapolis, MN), and monocyte chemoattractant protein-1 (MCP-1) (R&D Systems, Minneapolis, MN) were measured with corresponding ELISA kits following manufacturers’ instructions.

### Stroke model

To assess the effect of Psgl-1 deficiency on stroke in pristane-induced lupus mice, a stroke model induced by middle cerebral artery (MCA) occlusion was performed as previously described^12^. Briefly, after treatment with pristane for 32 weeks, animals were anesthetized with sodium pentobarbital (50 mg/kg, I.P.), and then a 4 mm vertical incision between the left ear and external canthus of the left eye was made. The temporal muscle was transected and the skull was exposed. The left MCA was visualized through the temporal bone and a 1.5-mW green light laser (540 nm, Melles Griot, Carlsbad, CA) was directed at the MCA from a distance of 6 cm before injection of rose Bengal (50 mg/kg in PBS) (Fisher, Fair Lawn, NJ) via the tail vein. The green light was directed to the MCA for 30 min, then the temporal muscle and skin was replaced. 72 hours after induction of thrombotic occlusion, mice were euthanized for stroke analysis.

To assess stroke volume, brain sections were stained with 4% 2,3,5-tri phenyltetrazolium chloride (TTC) as described^13^. Briefly, after euthanasia with pentobarbital, brains were removed and cut into 2 mm-thick coronal sections in a matrix (Harvard Apparatus, Holliston, MA). Sections were stained with 4% TTC in PBS for 20 min at 37 °C and fixed in 10% zinc formalin for 10 min. Viable tissue showed a red color after reaction with TTC while infarcted tissue was white. Images of 5 consecutive central sections of each brain were analyzed using Nikon MetaMorph software. The total infarct area of each brain was expressed as a percentage of total area of the ipsilateral hemisphere.

### Histology of kidneys

To examine the histology of kidneys after treatment with PBS or pristane, kidneys were fixed in 10% zinc formalin and embedded in paraffin. A series of 5 μm cross-sections containing the hilum of the kidney were stained with periodic acid-Schiff base (PAS) (Sigma, St Louis, MO). PAS-stained sections were scored as previously described^8^. Briefly, a semi-quantitative scoring system (0, no involvement; 0.5, minimal involvement of <10%; 1, mild involvement of 10–30%; 2, moderate involvement of 31–60%; 3, severe involvement of >60%) was used to assess 10 different parameters (mesangial hypercellularity, mesangial deposits, mesangial sclerosis, endocapillary cellular infiltrate, endocapillary sclerosis, endocapillary cellular crescents, endocapillary organized crescents, interstitial inflammation, tubular atrophy, and interstitial fibrosis). For glomerular indices, 30 glomeruli were examined and an average score was obtained. An activity and chronicity index was generated by compiling scores from groups of related parameters (for activity: mesangial hypercellularity, mesangial deposits, and endocapillary cellular infiltrate; for chronicity: endocapillary sclerosis, endocapillary organized crescents, tubular atrophy, and interstitial fibrosis).

### Immunohistochemistry

Neutrophils or macrophages in paraffin-embedded brain sections were identified with a rabbit anti-myeloperoxidase (MPO) polyclonal antibody (1:500) (DAKO, Carpinteria, CA) or a rat anti-mouse Mac-3 monoclonal antibody (1:200) (BD Biosciences, San Jose, CA) followed by detection with biotin-conjugated secondary goat anti-rabbit or goat anti-rat IgG (1:100) (Accurate Chemical & Scientific Corp., Westbury, NY). Stained cells were counted manually from five positively stained fields in each section using NIH ImageJ software and expressed as a percentage of total cells per field.

### Statistical analysis

All data are presented as mean ± standard error. Statistical analysis was carried out using GraphPad Prism. Results were analyzed using a 2-tailed t-test for comparisons between two groups. For multiple comparisons, results were analyzed using one-way ANOVA followed by Tukey post-test analysis. For evaluation of proteinuria, results were analyzed using the Mann-Whitney test. Probability values of p < 0.05 were considered statistically significant.

## Results and Discussion

Pristane has been widely used to induce a lupus-like syndrome in multiple mouse strains^14^,^15^. The leukocyte ligand Psgl-1 may promote inflammation in lupus by mediating leukocyte tissue infiltration through interaction with selectins. Genetic deficiency states of Psgl-1 and P-selectin have been previously studied in the MRL/MpJ-Fas^lpr^ model of lupus by performing intercrosses with mice deficient in P-selectin and Psgl-1. Surprisingly, mice deficient in P-selectin or Psgl-1 showed more rapid development of glomerulonephritis and dermatitis, along with earlier mortality^16^. Since regulation of inflammatory pathways may vary between different murine models of lupus and between different organs, we explored the role of Psgl-1 deficiency in mice with a homogenous strain background by administering pristane to WT and *Psgl-1*^−/−^ mice on the C57BL/6J genetic background. Since lupus is also characterized by premature stroke, which may be mediated by pathways independent of nephritis, we also designed the study to test the effect of Psgl-1 on stroke size following MCA occlusion.

### Effect of pristane treatment on auto-antibodies and nephritis in WT and *Psgl-1* ^−/−^ mice

**Figure 1:**
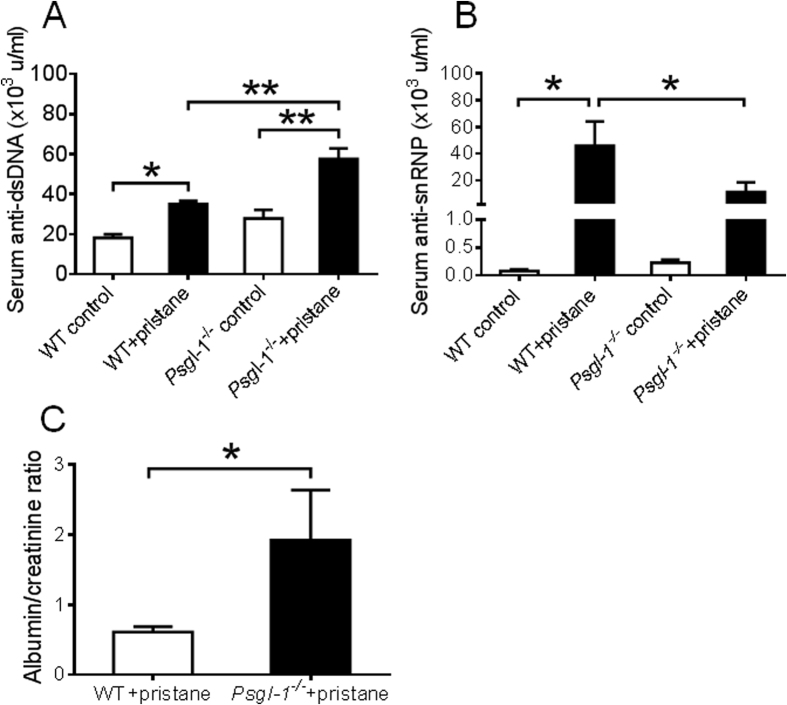
(**A**) Circulating levels of anti-dsDNA antibody in WT and *Psgl-1*^−/−^ mice treated with PBS or pristane for 32 weeks (n = 8 mice per group). (**B**) Circulating levels of anti-snRNP antibody in WT and *Psgl-1*^−/−^ mice treated with PBS or pristane for 32 weeks (n = 8 mice per group). (**C**) Urine albumin/creatinine ratios of WT (n = 18) and *Psgl-1*^−/−^ mice (n = 14) treated with pristane for 32 weeks. *P < 0.05. **P < 0.01.

Pristane has previously been shown to induce nephritis in C57BL/6J mice characterized by glomerular proliferation, mesangial expansion, and proteinuria^17^,^18^. These changes are associated with elevated levels of anti-dsDNA antibodies. Consistently, we observed increased anti-dsDNA antibodies in pristane-treated mice compared to PBS-treated mice (Fig. 1A), however, anti-dsDNA antibody levels were even higher in mice deficient in Psgl-1, suggesting that the Psgl-1 deficiency state may lead to enhanced production or reduced clearance of these antibodies. The levels of anti-snRNP were also significantly increased in WT mice treated with pristane compared to PBS-treated WT mice, however, in contrast to anti-dsDNA antibody levels, anti-snRNP induction by pristane was significantly attenuated in pristane-treated *Psgl-1*^−/−^ mice (Fig. 1B). These antibody patterns indicate that Psgl-1 deficiency may both promote and attenuate different autoimmune features of lupus.

**Figure 2:**
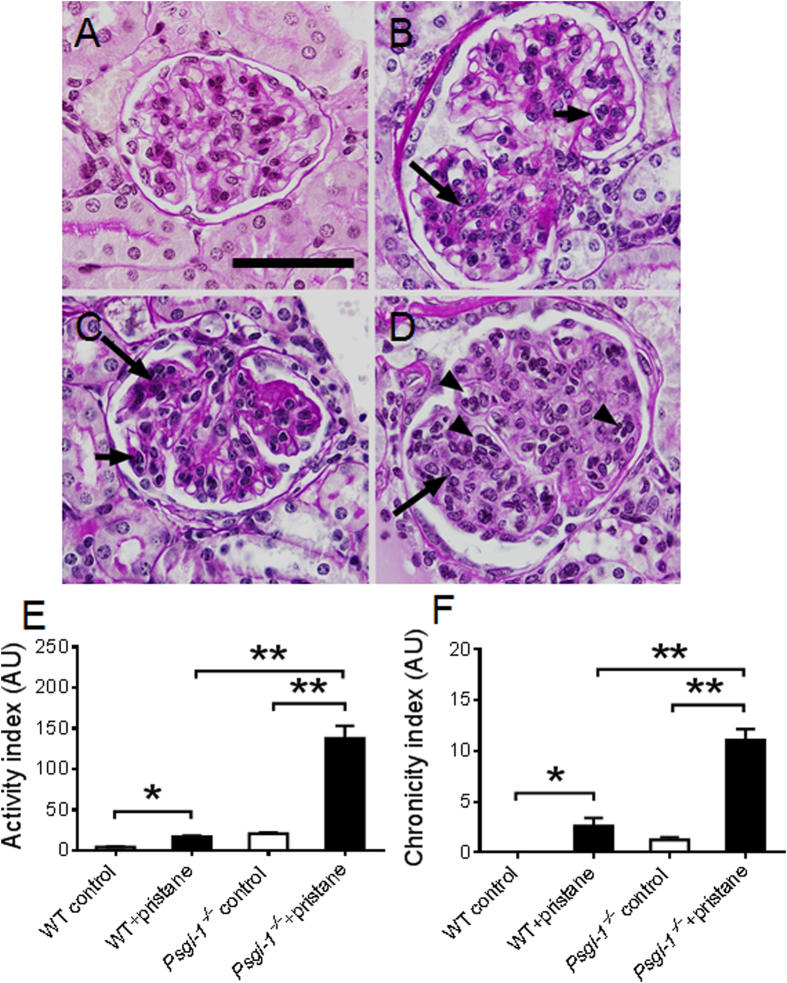
Glomerular histopathology in WT and *Psgl-1*^−/−^ mice treated with pristane or PBS for 32 weeks (n = 8 mice per group). (**A**–**D)** Representative glomeruli stained with Periodic Acid-Schiff (PAS) from control WT (**A**), pristane-treated WT (**B**), control *Psgl-1*^−/−^ (**C**), and pristane-treated *Psgl-1*^−/−^ mice (**D**). Glomeruli from pristane-treated WT (**B**) and control *Psgl-1*^−/−^ (**C**) mice demonstrated segmental increased mesangial and intracapillary hypercellularity from mesangial cell proliferation (long arrows) and single mononuclear leukocytes inside capillaries (short arrows). Most glomeruli from pristane-treated *Psgl-1*^−/−^ mice (**D**), however, showed global hypercellularity including mesangial cell proliferation (long arrow) and groups of intracapillary mononuclear leukocytes (arrowheads). (**E**) Activity index of PAS-stained sections calculated as described in Methods (n = 8 mice per group). (**F**) Chronicity index of PAS-stained sections calculated as described in Methods. *P < 0.05. **P < 0.01. Scale: 50 μm.

To determine the effect of Psgl-1 deficiency on nephritis in this model of lupus, kidney sections stained with PAS were scored by histopathology using both activity and chronicity indices that approximate the scoring system of human lupus nephritis. Both activity and chronicity were significantly increased in both pristane-treated WT and *Psgl-1*^−/−^ mice compared with their control groups, however, the indices were higher in pristane-treated *Psgl-1*^−/−^ mice compared with pristane-treated WT mice (Fig. 2). These findings indicate that similar to other lupus models, Psgl-1 deficiency exacerbates lupus nephritis following pristane administration. Consistently, the urine albumin-to-creatinine ratio was higher in pristane-treated *Psgl-1*^−/−^ mice compared to pristane-treated WT mice (Fig. 1C).

### Effect of pristane treatment on circulating inflammatory biomarkers and stroke in WT and *Psgl-1* ^−/−^ mice

A role for leukocyte extravasation may be especially prominent in the setting of acute macrovascular complications of lupus. Enhanced inflammatory responses to acute ischemic insults may lead to excessive end-organ damage in lupus^19^,^20^. A particularly devastating complication of lupus is premature stroke^21^. Cerebral leukocyte-endothelial interactions are increased in murine lupus models^22^ and soluble levels of adhesion molecules have been shown to correlate with disease activity and prognosis^23^ suggesting leukocyte adhesive interactions may promote some types of end organ damage.

**Figure 3:**
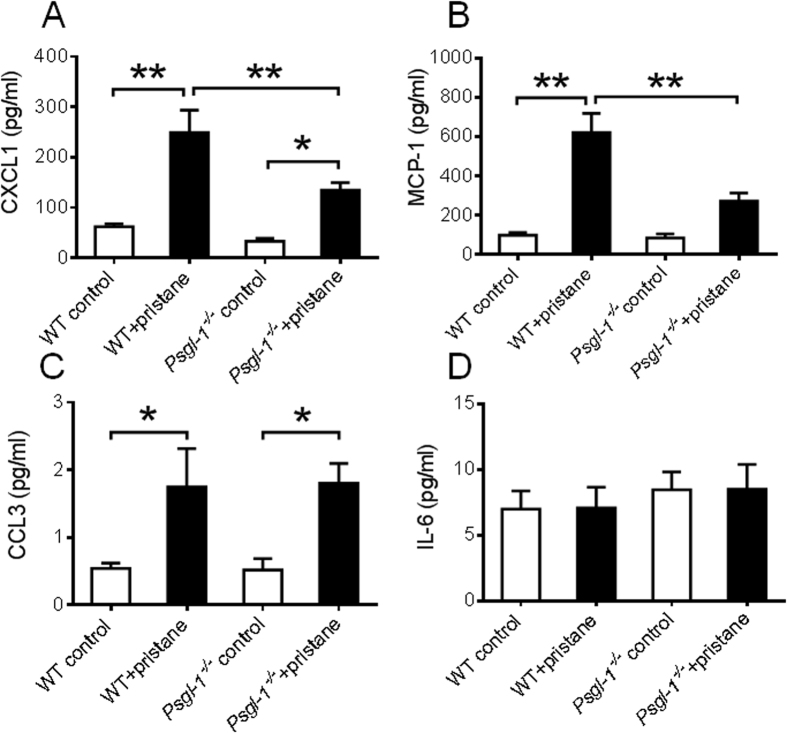
Measurements of circulating biomarkers in WT and *Psgl-1*^−/−^ mice treated with PBS or pristane for 32 weeks (n = 8 mice per group). (**A**) Levels of chemokine (C-X-C motif) ligand 1 (CXCL1). (**B**) Levels of monocyte chemoattractant protein-1 (MCP-1). (**C**) Levels of chemokine (C-C motif) ligand 3 (CCL3). (**D**) Levels of interleukin-6 (IL-6). *P < 0.05. **P < 0.01.

To determine the effect of pristane on a circulating inflammatory biomarkers potentially involved in leukocyte recruitment, the plasma levels of CXCL1, IL-6, CCL3, and MCP-1 were measured in PBS-treated or pristane-treated mice. 32 weeks after treatment, levels of CXCL1 (Fig. 3A), MCP-1 (Fig. 3B), and CCL3 (Fig. 3C) were significantly increased in WT mice treated with pristane compared to PBS-treated WT mice, however, CXCL1 and MCP-1 induction by pristane was significantly attenuated in pristane-treated *Psgl-1*^−/−^ mice (Fig. 3A,B). No difference was detected in levels of CCL3 between WT and *Psgl-1*^−/−^ mice after pristane treatment (Fig. 3C). Levels of CXCL1, MCP-1, or CCL3 were similar between control WT and *Psgl-1*^−/−^ mice treated with only PBS while no differences were detected in levels of IL-6 among these groups with or without pristane treatment (Fig. 3D).

**Figure 4:**
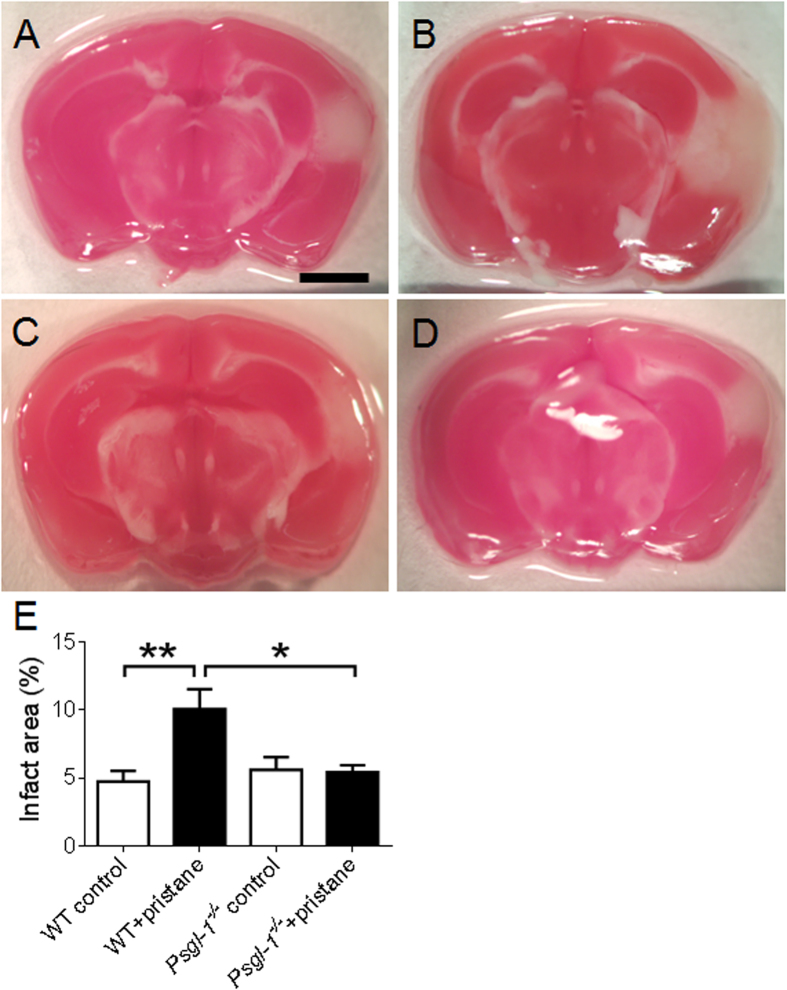
Ischemic stroke in WT and *Psgl-1*^−/−^ mice treated with PBS or pristane for 32 weeks (n = 8 mice per group). (**A**–**D)** Representative brain slides stained with TTC from control WT (**A**), pristane-treated WT (**B**), control *Psgl-1*^−/−^ (**C**), and pristane-treated *Psgl-1*^−/−^ mice (**D**). (**E**) Quantification of infarct area expressed as a percentage of total area of ipsilateral hemisphere. *P < 0.05. **P < 0.01. Scale: 2 mm.

To determine if pristane-treated *Psgl-1*^−/−^ mice were protected from acute tissue injury, the effect of Psgl-1 deficiency in a model of acute ischemic stroke was determined. Stroke was induced by photochemical-mediated injury to the MCA which leads to sustained thrombotic occlusion of the MCA^13^,^24^. Following MCA occlusion, the infarct area was significantly larger in pristane-treated WT mice compared to PBS-treated WT mice (Fig. 4) (additional images in Supplemental Fig. 1). However, compared to pristane-treated WT mice, the infarct area in pristane-treated *Psgl-1*^−/−^ mice was significantly reduced (Fig. 4) while no difference was detected between PBS-treated control WT and control *Psgl-1*^−/−^ mice (Fig. 4). Since hypertension is strongly associated with stroke morbidity, blood pressure was also measured. Stroke size was not affected by systemic blood pressure as no changes in systolic blood pressure were observed following pristane treatment between the groups (systolic blood pressure: 112.1 ± 5.5 mmHg for WT and 109.3 ± 7.5 mmHg for *Psgl-1*^−/−^ mice).

### Neutrophil and macrophage infiltration after stroke

**Figure 5:**
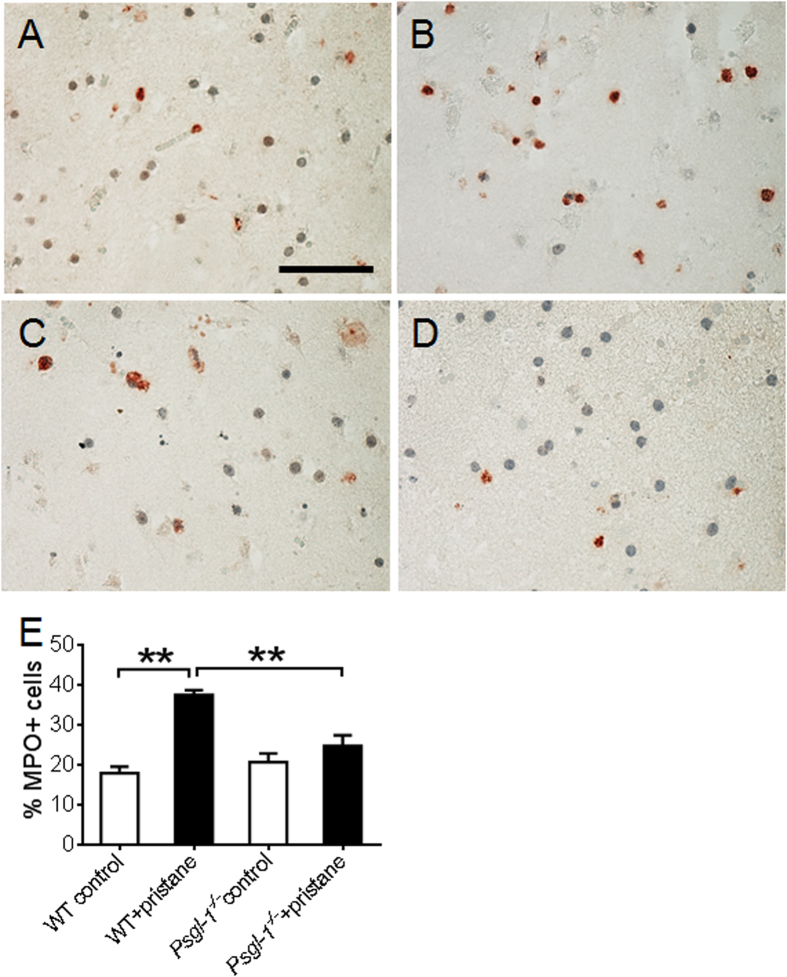
Neutrophil infiltration after stroke examined by myeloperoxidase (MPO) staining of cerebral infarct area from WT and *Psgl-1*^−/−^ mice treated with PBS or pristane for 32 weeks (n = 8 mice per group). (**A**–**D**) Representative photomicrographs of MPO staining in brain cross sections from control WT (**A**), pristane-treated WT (**B**), control *Psgl-1*^−/−^ (**C**), and pristane-treated *Psgl-1*^−/−^ mice (**D).** (**E**) Quantification of MPO-positive cells at infarct area. **P < 0.01. Scale: 50 μm.

**Figure 6:**
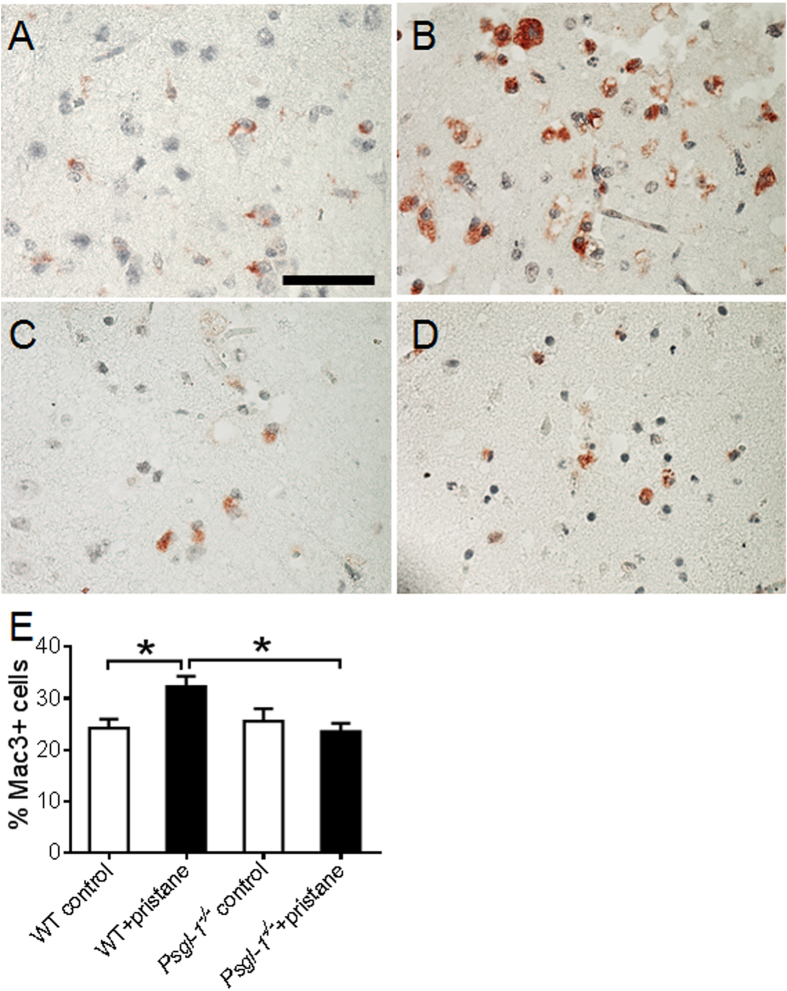
Macrophage infiltration after stroke examined by Mac-3 staining at cerebral infarct area of WT and *Psgl-1*^−/−^ mice treated with PBS or pristane for 32 weeks (n = 8 mice per group) . (**A**–**D)** Representative photomicrographs of Mac-3 staining in brain cross sections from control WT (**A**), pristane-treated WT (**B**), control *Psgl-1*^−/−^ (**C**), and pristane-treated *Psgl-1*^−/−^ mice (**D**). (**E**) Quantification of Mac-3-positive cells at infarct area. *P < 0.05. Scale: 50 μm.

Ischemic stroke is characterized by increased inflammatory cell infiltration, which exacerbates cerebral infarction^25^. To assess inflammatory cell recruitment in lupus mice after stroke, neutrophils were detected 3 days following stroke induction. Neutrophil infiltration was significantly increased in the infarct area of pristane-treated WT mice compared to PBS-treated WT mice (Fig. 5), while neutrophil accumulation was reduced in pristane-treated *Psgl-1*^−/−^ mice compared to pristane-treated WT mice (Fig. 5). Neutrophil infiltration was similar between PBS-treated WT and PBS-treated *Psgl-1*^−/−^ mice (Fig. 5). Macrophage detection in the cerebral infarct area was similarly increased in pristane-treated WT mice compared to PBS-treated control WT mice, while the macrophage infiltration was significantly reduced in pristane-treated *Psgl-1*^−/−^ mice compared with pristane-treated WT mice (Fig. 6). There was no difference in macrophage accumulation between PBS-treated control WT mice and PBS-treated *Psgl-1*^−/−^ mice (Fig. 6).

In conclusion, Psgl-1 deficiency is associated with reduced stroke size in a murine model of lupus, despite exacerbation of nephritis. Pathways involved in cerebral damage following ischemic insult may therefore differ from those involved in lupus nephritis and may require different treatment strategies. For example, Psgl-1 deficiency may simultaneously impair the recruitment of a protective lymphocyte population to the kidney while also reducing accumulation of tissue destructive neutrophils and macrophages at the site of acute ischemic injury. Targeting molecules involved in leukocyte adhesion may thus be particularly effective in acute vascular complications of lupus. Elucidation of pathways downstream of Psgl-1 will aid in developing therapies that may prevent macrovascular complications of lupus without promoting nephritis.

## Additional Information

**How to cite this article**: Wang, H. *et al.* Psgl-1 Deficiency is Protective against Stroke in a Murine Model of Lupus. *Sci. Rep.* **6**, 28997; doi: 10.1038/srep28997 (2016).

## Supplementary Material

Supplementary Information
