# Supplementary material for: Psgl-1 Deficiency is Protective against Stroke in a Murine Model of Lupus
Source: Sci Rep. 2016 Jun 30;6:28997. doi: 10.1038/srep28997 (PMC4928054; doi:10.1038/srep28997)
Supplement: Supplementary Information [file srep28997-s1.pdf]

Psgl-1 Deficiency is Protective against Stroke in a Murine Model of Lupus

Short title: Psgl-1 and stroke in lupus

Hui Wang[1], Jason S. Knight[2], Jeffrey B. Hodgin[3], Jintao Wang[1], Chiao Guo[1], Kyle Kleiman[1], Daniel T. Eitzman [1*]

1 University of Michigan, Department of Internal Medicine, Cardiovascular Research Center, Ann Arbor, Michigan, USA

2 University of Michigan, Department of Internal Medicine, Division of Rheumatology, Ann Arbor, Michigan, USA

3 University of Michigan, Department of Pathology, Ann Arbor, Michigan, USA

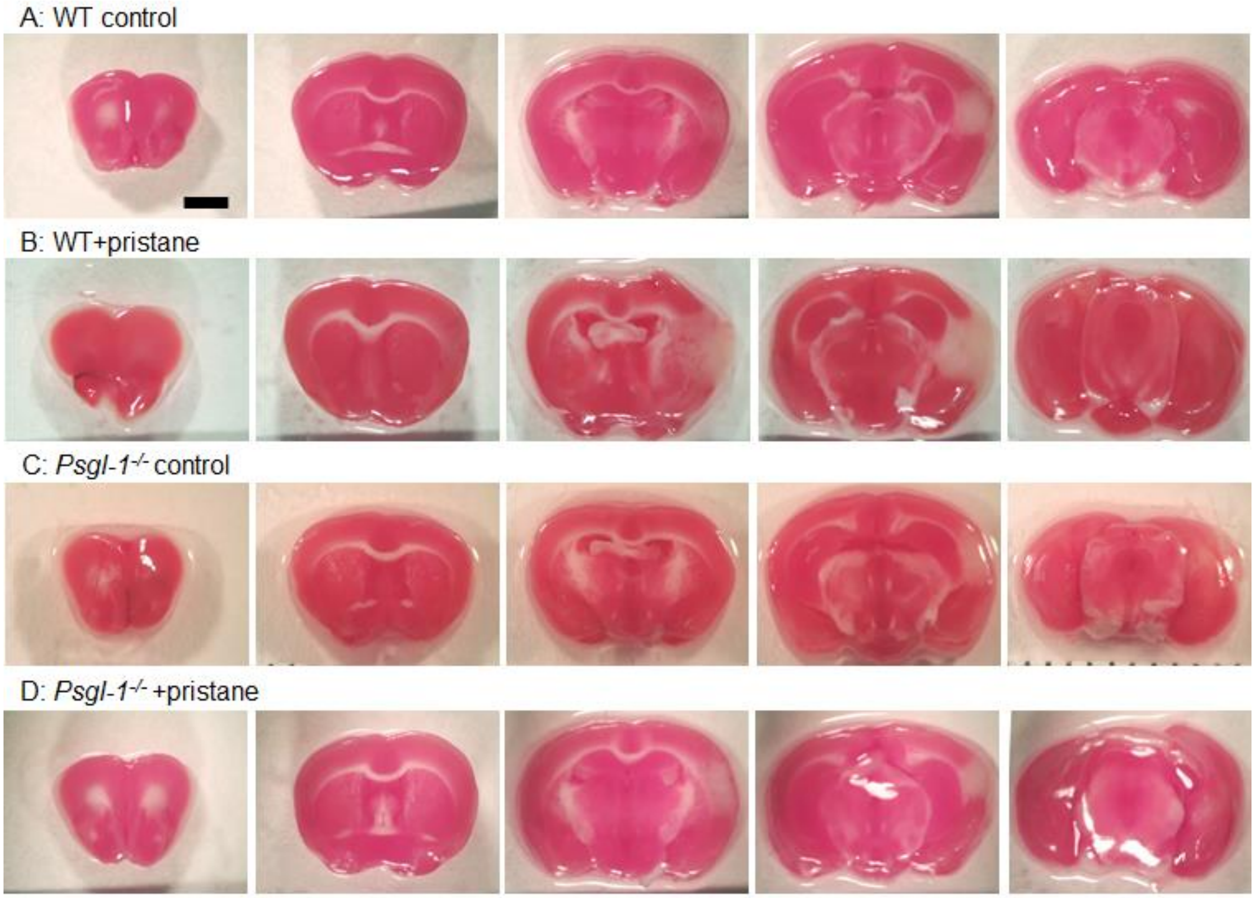

Supplemental Figure 1. Representative sections of brain after stroke stained with TTC. A, B, C, D: Serial rostral (left) to caudal (right) coronal sections of brain slides from control WT (A), pristane-treated WT (B), control *Psgl-1*$^{-/-}$ (C), and pristane-treated *Psgl-1*$^{-/-}$ mice (D). Scale: 2mm.
